# Unklare Rundherde und Lymphadenopathie – die Spur führt zur Natur

**DOI:** 10.1007/s00108-025-01895-4

**Published:** 2025-05-05

**Authors:** Philipp Gessner, Maximillian Von Laffert, Sebastian Ullrich, Hubert Wirtz, Hans-Jürgen Seyfarth

**Affiliations:** 1 Klinik und Poliklinik für Onkologie, Gastroenterologie, Hepatologie und Pneumologie, Universitätsklinikum Leipzig, Liebigstr. 20, 04103 Leipzig, Deutschland; 2 Institut für Pathologie, Universitätsklinikum Leipzig, Leipzig, Deutschland; 3 Klinik und Poliklinik für Diagnostische und Interventionelle Radiologie, Universitätsklinikum Leipzig, Leipzig, Deutschland

**Keywords:** Tularämie, Pneumonie, Franciscella tularensis, Zoonotische Infektionen, Mediastinale Lymphadenopathie, Tularemia, Pneumonia, Franciscella tularensis, Zoonosis, Mediastinal lymphadenopathy

## Abstract

Wir berichten über zwei Patienten, die im Jahr 2023 mit pulmonalen Herdbefunden und mediastinaler Lymphknotenschwellung im Universitätsklinikum Leipzig behandelt wurden. Histologisch zeigte sich in beiden Fällen eine granulomatöse Entzündungsreaktion. Als Ursache der Erkrankung konnte eine Infektion mit *Francisella tularensis* nachgewiesen werden. Für beide Patienten bestand die mutmaßliche Infektionsquelle in der freizeitlichen Beschäftigung mit der Jagd, die eines der Risiken für eine Infektion darstellt. Unter antibiotischer Therapie boten beide Fälle einen unkomplizierten Verlauf.

## Abstract (en)

We report on two patients with pulmonary lesions and mediastinal lymph node enlargement who were treated in 2023 at University of Leipzig Medical Center. Histologically, both cases showed a granulomatous inflammatory reaction. As the cause of the disease an infection with *Francisella tularensis *could be detected. For both patients the source of infection was assumed to be a history of recreational hunting, which represents a risk for infection. Under antibiotic therapy both cases showed an uncomplicated course.

## Fall 1

### Anamnese

Ein 61-jähriger Patient ohne bekannte Vorerkrankungen stellte sich initial in der Notaufnahme mit einer symptomatischen Episode eines neu aufgetretenen Vorhofflimmerns vor. Erst im Verlauf traten am 4. stationären Tag Fieber (bis 39,4 °C) und Dyspnoe mit respiratorischer Insuffizienz (Sauerstoffpartialdruck [pO_2_] 65,5 mm Hg, Kohlenstoffdioxidpartialdruck [pCO_2_] 27,5 mm Hg unter Raumluft) auf.

### Untersuchung

Auskultatorisch bot der Patient ein vesikuläres Atemgeräusch, jedoch keine Nebengeräusche. In der Perkussion konnte kein pathologischer Befund erhoben werden. Passend zur respiratorischen Insuffizienz zeigte der Patient eine Tachypnoe (Atemfrequenz 20/min) als Zeichen einer erhöhten Atemanstrengung.

### Initiale Diagnostik

**Abb. 1:**
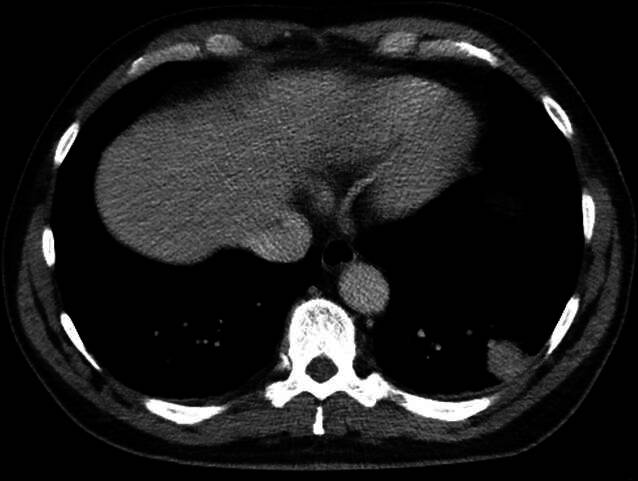
Ca. 2,5 × 2,4 cm messender subpleuraler Rundherd im linken Lungenunterlappen – malignomsuspekt, DD nachrangig Rundatelektase bzw. entzündlich-infiltrativ. Zusätzlich, hier nicht abgebildet, deutlich vermehrte und teils pathologisch vergrößerte Lymphknoten mediastinal sowie links hilär

Begleitend zu den respiratorischen Beschwerden beobachteten wir einen Anstieg der Entzündungswerte (Leukozyten 14,4^9^/l, CRP 180 mg/l). Unter dem Verdacht auf einen pulmonalen bzw. mediastinalen Fokus führten wir, aufgrund des deutlich beeinträchtigten Zustands des Patienten, eine Computertomographie (CT) des Thorax durch. Dabei fielen linksseitig eine keilförmige, rundherdartige Konsolidierung dorsobasal sowie eine mediastinale Lymphadenopathie auf (Abb. 1). Aufgrund des plötzlichen Auftretens der Dyspnoe, eines Wells-Scores I von 4,5 Punkten (Herzfrequenz > 100/min, pulmonale Embolie erschien als wahrscheinlichste Ursache) und eines D‑Dimers von 1,45 mg/l bestand weiterhin der Verdacht auf eine periphere Lungenarterienembolie. Durch die CT des Thorax in venöser Kontrastmittelphase konnte der Verdacht nicht ausgeräumt werden. Aufgrund der vermuteten peripheren Lage der Embolie wurde eine Perfusions‑/Ventilationsszintigraphie ergänzt. Hierbei konnte kein Perfusions‑/Ventilations-Mismatch als Korrelat einer Lungenarterienembolie detektiert werden.

### Therapie und Verlauf

Aufgrund des plötzlichen Auftretens der Symptome in Kombination mit den CT-Befunden wurde unter dem Verdacht auf eine nosokomial erworbene Pneumonie eine kalkulierte antibiotische Therapie mit Piperacillin/Tazobactam begonnen. Eine Sputumuntersuchung, Blutkulturanalysen sowie eine PCR-Untersuchung auf respiratorische Viren waren ohne wegweisendes Ergebnis. Unter der antibiotischen Therapie kam es jedoch zu keiner Besserung, sodass die Anamnese noch einmal vertieft wurde. In diesem Zusammenhang berichtete der Patient, in seiner Freizeit zu jagen. Dabei habe er vor ein bis zwei Wochen einen kürzlich verendeten Hasen für die Hundeausbildung genutzt. In diesem Kontext ergab sich nun der Verdacht auf eine Tularämie, weswegen die antibiotische Therapie auf Levofloxacin umgestellt wurde.

Im Sinne der Verdachtsdiagnose konnten serologisch deutlich erhöhte IgG-/IgM-Titer für *Francisella tularensis* (IgG-Ak 123,8 U/ml; IgM-Ak 24,1 U/ml) gefunden werden.

Unter der umgestellten antibiotischen Therapie konnte eine zügige Besserung der Symptomatik mit Regredienz der laborchemischen Inflammationsparameter beobachtet werden.

**Abb. 2:**
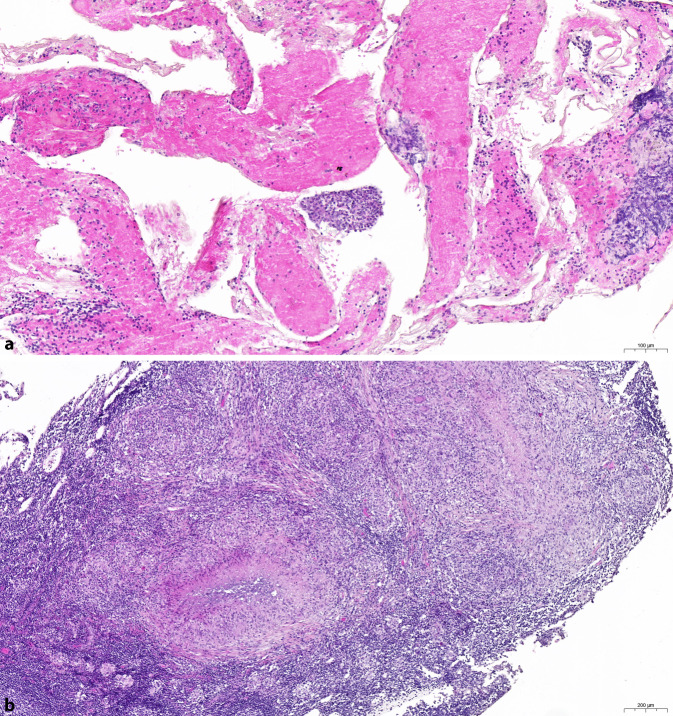
**a** *Histologie* *1:* LK-Station IV mit Nachweis von neutrophilen Granulozyten, partieller Nekrose, fokalen Histiozyten und abschnittsweiser herdförmiger granulomatöser Entzündungsreaktion. **b** *Histologie* *2:* Lymphknotenexstirpate Station VII mit Nachweis einer epitheloidzellig-granulomatösen, nekrotisierenden Entzündung

Zur weiteren Untermauerung der Verdachtsdiagnose und differenzialdiagnostischen Abklärung führten wir, bei in der Computertomographie auffälliger mediastinaler Lymphadenopathie, eine Bronchoskopie mit endobronchialer Sonographie (EBUS) mit transbronchialer Nadelaspiration (TBNA) der Lymphknotenstation 4 durch. Histologisch präsentierten sich eine partielle Nekrose, fokale Histiozyten und eine kleinherdige, sehr fokale granulomatöse Entzündungsreaktion. Führend war eine durch neutrophile Granulozyten geprägte, also floride Entzündungskomponente (Abb. 2a). Eine PCR-Untersuchung auf *M. tuberculosis* erbrachte ein negatives Ergebnis. Parallel konnte die Verdachtsdiagnose durch den Nachweis von *Francisella-tularensis*-spezifischer Nukleinsäure durch Polymerase-Kettenreaktion (PCR) bestätigt werden (Subspezies-Differenzierung: *Francisella tularensis holarctica*).

**Abb. 3:**
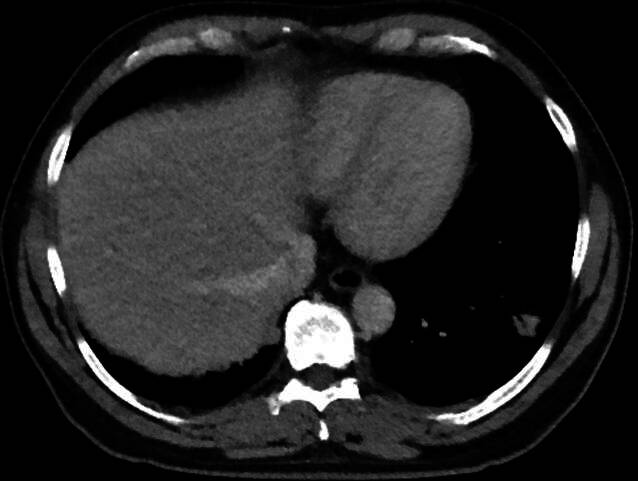
Im Vergleich zur VU (Abb. 1) deutlich größenregrediente Darstellung der subpleuralen, rundherdartigen Konsolidierung dorsolateral im linken Unterlappen mit Residualbefund a. e. (post-)entzündlicher Genese. Ebenfalls deutlich regrediente Darstellung der vormals vermehrten sowie vergrößerten Lymphknoten mediastinal sowie links hilär

Die CT-Verlaufsuntersuchung nach 6 Wochen zeigte einen rückläufigen Befund (Abb. 3).

## Fall 2

### Anamnese

Ein 46-jähriger Patient ohne Nikotinanamnese stellte sich mit Verdacht auf ein Lungenkarzinom rechts zentral mit mediastinaler Lymphknotenmetastasierung vor.

Ambulant hatten ein erhöhtes D‑Dimer in der Kombination mit einem Gewichtsverlust von 12 kg in einem Zeitraum von ca. drei Monaten, Abgeschlagenheit und Fieberschübe bis 41 °C zu einer Computertomographie des Thorax mit Kontrastmittel geführt. Im ambulanten Setting trat unter antipyretischer Therapie und antibiotischer Vorbehandlung mit Penicillin- und Makrolidantibiotika keine Besserung ein. Erst die empirische Gabe von Doxycyclin 200 mg über 14 Tage führte zur Entfieberung und Besserung der Beschwerden. Drei Tage nach Beendigung der antibiotischen Therapie stellte sich der Patient in unserer Klinik vor.

### Untersuchung

In der körperlichen Untersuchung sahen wir einen Patienten in gutem Allgemeinzustand mit normosomem Ernährungszustand. In der klinischen Untersuchung ließ sich ein vesikuläres Atemgeräusch auskultieren, in der Perkussion war ein sonorer Klopfschall zu vernehmen. Darüber hinaus verblieb auch der übrige körperliche Status ohne pathologischen Befund.

### Diagnostik

**Abb. 4:**
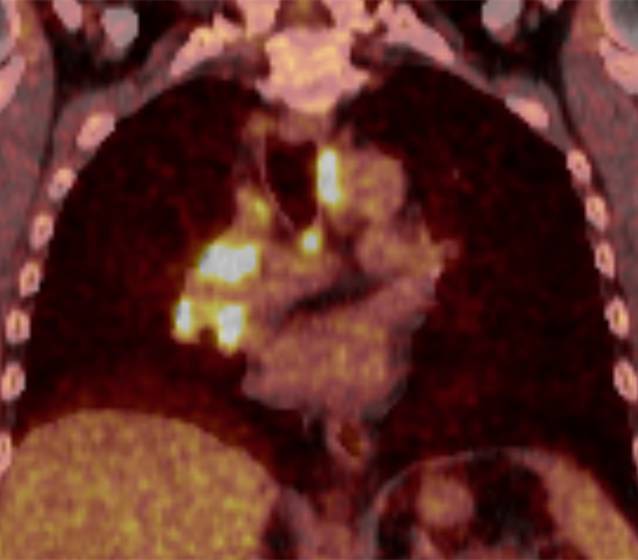
Malignitätssupekt gesteigerte Glukoseutilisation rechts hilär/zentral (SUV_max_ 18,2 und 15,0) sowie in den mediastinalen Lymphknoten bis links infraklavikulär

Unter dem Verdacht auf ein lokal begrenztes Tumorleiden führten wir zur Lymphknotendiagnostik und zum Ausschluss von Fernmetastasen eine Positronenemissionstomographie-CT (PET-CT) durch. Die pulmonalen Herdbefunde sowie die mediastinalen Lymphknoten wiesen eine ausgeprägte Glukoseavidität auf (Abb. 4). Ein Hinweis auf eine Fernmetastasierung ergab sich aus dieser Untersuchung nicht. Zur histologischen Sicherung und Festlegung des Lymphknoten-Stagings erfolgte ein endobronchialer Ultraschall mit transbronchialer Nadelaspiration. Zytologisch zeigte sich repräsentatives Lymphknotenmaterial ohne Hinweis auf maligne Tumorzellen. Die daher zur weiteren Abklärung durchgeführte Mediastinoskopie zeigte im Bereich der Lymphknoten-Station 7 deutlich vergrößerte und vulnerable Strukturen. Auf Druck entleerte sich reichlich Eiter. Histologisch fand sich eine epitheloidzellig-granulomatöse, nekrotische Entzündung mit diskontinuierlich eingestreuten Langhans-Riesenzellen ohne den Nachweis von säurefesten Stäbchen (Abb. 2b). Laborchemisch bestand eine geringgradige Erhöhung des C‑reaktiven Proteins (24,3 mg/l) ohne Leukozytose. Der Enzyme-Linked ImmunoSpot (ELISpot) verblieb ohne Reaktivität auf *M. tuberculosis*.

### Therapie und Verlauf

Nunmehr mit dem Verdacht auf eine infektiöse Genese befragten wir den Patienten erneut. Es konnte herausgearbeitet werden, dass wenige Tage nach Erlegung und Zerwirkung eines Wildschweins akut Fieber bis 41 °C und Schüttelfrost aufgetreten seien. Hieraus ergab sich schließlich der Verdacht auf eine Infektion mit *Francisella tularensis*.

Serologisch ließ sich ein positiver Screeningtest auf *Francisella tularensis* erheben. Ein Bestätigungstest wies einen IgG-Antikörper-Wert von > 300 U/ml sowie einen IgM-Antikörper-Wert von > 500 U/ml auf. Zur Festigung der Verdachtsdiagnose veranlassten wir aus den intraoperativen Abstrichen der nekrotisch zerfallenden Lymphknoten weitere Diagnostik. Durch PCR konnte *Francisella-tularensis*-spezifische Nukleinsäure nachgewiesen werden (Subspezies-Differenzierung: *Francisella tularensis holarctica*). In Anbetracht des weiterhin deutlich erhöhten IgM-Titers und des ausgeprägten lokalen Befunds etablierten wir erneut eine Doxycyclintherapie mit 200 mg einmal täglich für 7 Tage.

**Abb. 5:**
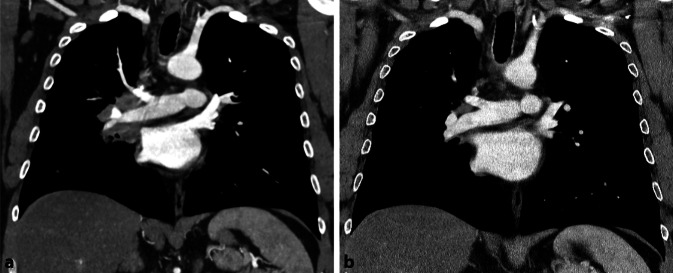
**a** Rechts zentrale Raumforderung mit Infiltration des Mediastinums sowie Kompression des bronchovaskulären Bündels des rechten Mittel- und Unterlappens. Vermehrte und vergrößerte mediastinale Lymphknoten. **b** In der Verlaufskontrolle deutliche Regredienz der pulmonalen Raumforderung rechts zentral mit geringem Residualbefund a. e. (post-)entzündlicher Genese

In der CT-Verlaufsbildgebung nach 4 Monaten ließ sich eine Größenregredienz der pulmonalen Raumforderung verzeichnen (Abb. 5). Neue Beschwerden traten bei dem Patienten nicht mehr auf.

## Gemeinsame Diskussion

Tularämie, auch als Hasenpest bekannt, ist eine bakterielle Zoonose, die durch das fakultativ intrazellulär lebende, gramnegative Bakterium *Francisella tularensis* ausgelöst wird [1]. Von den vier bekannten Subspezies sind insbesondere *F. tularensis *ssp.* tularensis* (Biovar Typ A), die vorwiegend in Nordamerika vorkommt, und *F. tularensis *ssp.* holarctica* (Biovar Typ B), die weltweit in der nördlichen Hemisphäre verbreitet ist, von klinischer Bedeutung. Bisher ist *F. tularensis *ssp. *holarctica* die einzige bekannte Subspezies, die in Deutschland nachgewiesen wurde [2]. Die Übertragung von Tularämie auf den Menschen erfolgt in der Regel durch den Kontakt mit erkrankten Tieren, wobei insbesondere Kleinnager als Erregerreservoir fungieren. Aber auch Arthropoden wie Zecken, Mücken und Fliegen können als Vektoren dienen [1]. In Deutschland stellt die Tularämie eine seltene Erkrankung mit tendenziell steigender Inzidenz dar. Im Jahr 2022 wurden deutschlandweit 68 Erkrankungsfälle sowie ein Todesfall beim RKI gemeldet. Obwohl es im Vergleich zum Jahr 2021 zu einer Abnahme der Erkrankungsfälle kam, zeigt sich in den letzten 10 Jahren eine stetige Zunahme der gemeldeten Erkrankungsfälle [3]. Meist treten wenige Tage nach Exposition unspezifische grippale Symptome wie Allgemeinzustandsverschlechterung, Fieber, Schüttelfrost oder Lymphadenopathie auf. Die weitere klinische Präsentation unterscheidet, in Abhängigkeit vom Übertragungsweg, unterschiedliche Verlaufsformen. Exemplarisch liegt bei der ulzeroglandulären Form neben einer lokal ulzerierenden Entzündungsreaktion, meist im Bereich des Insektenstichs, eine reaktive Lymphknotenschwellung vor. Bei der glandulären Form fehlt die Ausbildung einer lokalen Ulzeration. Eine oropharyngeale Verlaufsform ist nach oraler Aufnahme von kontaminiertem Wasser oder Lebensmitteln möglich. Hierbei findet man eine zervikale bzw. submandibuläre Lymphknotenschwellung mit Pharyngitis oder Stomatitis, gegebenenfalls mit Auftreten von gastrointestinalen Begleitbeschwerden [4, 5]. In beiden Fallbeispielen beobachteten wir die seltener vorkommende pulmonale Verlaufsform der Tularämie, die nach dem Umgang mit betroffenen Wildtieren auftreten kann [4].

Beide Fälle demonstrieren eindrucksvoll, dass bei pulmonalen Herdbefunden mit mediastinalen Lymphknotenschwellungen neben den häufigeren Erkrankungen, wie Lungenkarzinom, Sarkoidose oder der pulmonalen Tuberkulose, auch seltenere infektiologische Erkrankungen in Betracht gezogen werden sollten.

In Deutschland wird eine Seroprävalenz von 0,2 bis 1,7 % beschrieben [6], sodass eine serologische Untersuchung eine sinnvolle Ergänzung bei Verdacht auf Tularämie darstellt. Eine Subspezifizierung von *F. tularensis* anhand der Serologie ist nicht möglich.

Die Diagnose der Tularämie kann häufig durch eine Kombination aus zielgerichteter Anamneseerhebung in Zusammenschau mit den serologischen und gegebenenfalls histologischen Ergebnissen gestellt werden. Die histologische Darstellung kann dabei vielfältig sein und reicht von einer unspezifischen, floriden sowie retikulohistiozytär-abszedierenden Entzündung bis hin zu Granulomen mit oder ohne Nekrose [7].

Insbesondere bei pulmonalen Manifestationen mit differenzialdiagnostischem Verdacht auf eine Pneumonie, wie im ersten beschriebenen Fall, ist zu beachten, dass eine antibiotische Therapie mit Penicillinen oder anderen Beta-Laktam-Antibiotika wirkungslos ist [8]. Bei Hinweis darauf, dass eine Infektion mit *F. tularensis* als möglicher Erreger in Betracht gezogen werden müsste, sollte eine Therapieumstellung auf Fluorchinolone oder Doxycyclin erfolgen. Prospektive und randomisierte Daten zum Vergleich verschiedener Behandlungsregime liegen nicht vor. Obwohl die meisten Daten zur Therapie über Ciprofloxacin vorliegen, stellte in unserem Fall Levofloxacin 500 mg zweimal täglich eine gute Alternative dar [9].

Bei PET-positiven Raumforderungen in der Lunge muss primär an ein malignes Geschehen gedacht werden. Trotz des untypischen Verlaufs mit Fieber erschien ein primäres Lungenkarzinom zunächst als wahrscheinlichste Diagnose. Erst der Operationsbefund der Mediastinoskopie mit histologischem Nachweis von Granulomen lenkte unseren Fokus auf eine infektiologische Ursache. In Fallbeispielen wird eine Antibiose von insgesamt 21 Tagen nach Materialgewinnung mittels Punktion oder Biopsie empfohlen, sodass wir eine prolongierte antibiotische Therapie mit Doxycyclin ergänzten [10]. Der mutmaßliche Infektionsmechanismus, sich beim Zerwirken eines Wildschweins angesteckt zu haben, unterstreicht die Kontagiosität des Erregers, insbesondere bei Kontakt zu Wildtieren. Bereits kleinste Aerosolbildung mit der Inhalation von 25 Bakterien aus den Körpern infizierter Tiere, im Rahmen der Fleischverarbeitung oder bei Kontakt zu verendeten Tieren kann zur Infektion führen [11]. Gemäß Infektionsschutzgesetz § 7 Abs. 1 besteht eine namentliche Meldepflicht bei direktem oder indirektem Nachweis, sofern dieser auf eine akute Infektion hinweist. Eine Übertragung von Mensch zu Mensch wurde bisher noch nicht beschrieben. Isolationsmaßnahmen für betroffene Patienten und Patientinnen sind daher nicht notwendig.

Die Diagnose der Tularämie lässt sich aus Anamnese, klinischem Erscheinungsbild und serologischen Untersuchungen stellen. Molekularbiologische Untersuchungen können hilfreich sein, die Diagnose zu festigen.

In der Regel ist eine Therapie mit Ciprofloxacin 500 mg zweimal täglich über 10 bis 14 Tage oder eine Therapie mit Doxycyclin 200 mg täglich für 14 bis 21 Tage ausreichend. Bei schweren Verläufen ist eine Kombination aus Gentamicin 5 mg/kgKG i.v. und Ciprofloxacin 500 mg zweimal täglich über 10 bis 14 Tage empfohlen [8].

Die Tularämie ist eine wichtige Infektionskrankheit, an die differenzialdiagnostisch, wegen steigender Inzidenz, bei passender Anamnese und bei mediastinaler Lymphadenopathie gedacht werden sollte.

## Fazit für die Praxis

- Eine Isolation betroffener Patienten und Patientinnen muss nicht erfolgen.
- Bei Verdacht auf Tularämie sollte eine antibiotische Therapie mit Fluorchinolonen oder Doxycyclin erfolgen.
- Gefährdeten Personengruppen, wie zum Beispiel Jägern und Jägerinnen, sollte man, wenngleich seitens des Deutschen Jagdverbands keine generalisierte Empfehlung besteht, beim Umgang mit Wildtieren zum Tragen von Schutzhandschuhen und einer Schutzmaske (z. B. FFP2) raten.
